# Cross-cultural adaptation and validation of the Greek version of the Kerlan-Jobe orthopaedic clinic shoulder and elbow score in Greek overhead athletes

**DOI:** 10.12688/f1000research.134195.1

**Published:** 2023-05-15

**Authors:** Eleftherios Paraskevopoulos, George Plakoutsis, Maria Papandreou

**Affiliations:** 1 Department of Physiotherapy, School of Health Sciences, Panepistemio Peloponnesou, Tripoli, Peloponnese, Greece; 2 Department of Physiotherapy, Panepistemio Dytikes Attikes, Egaleo, Athens, Greece

**Keywords:** Overhead athlete, questionnaire, patient-reported outcome, elbow, shoulder, Kerlan-Jobe Orthopedic Clinic

## Abstract

**Background:** Overhead athletes frequently perform rapid and powerful throwing overhead strokes in positions at the extreme range of motion, increasing the risk of upper limb injury. The Kerlan-Jobe Orthopedic Clinic (KJOC) Shoulder and Elbow Score has shown to be a valid and reliable questionnaire that can be used for the assessment of the functional status of the upper limb of patients involved in highly demanding overhead sports. The KJOC has been translated into several other languages however, a Greek version of the KJOC is not available yet.

**Methods:** The KJOC will be cross-culturally adapted into Greek following international guidelines. At least 100 overhead athletes with or without shoulder or elbow complaints will be recruited and asked to fill in the Greek version of the KJOC twice and the Disabilities of Arm, Shoulder and Hand Questionnaire (DASH) once. The internal consistency and the test-retest reliability will be examined using Cronbach’s alpha and the intraclass correlation coefficient (ICC), respectively. The standard error of measurement (SEM) and the minimum detectable change (MDC) will be calculated and possible ground or ceiling effects will be also examined. Convergent validity will be evaluated with the Greek DASH using Pearson’s correlation.

**Results:** The results of this study will be presented in an article to be published later.

**Conclusions:** This report describes the process of translation and cross cultural adaptation of the Greek version of the KJOC. We believe a study protocol will assist researchers in the field to improve the reporting of similar studies and as a result improve the quality of their studies.

## Introduction

It is well known that overhead athletes frequently perform rapid and powerful throwing strokes above head-height during ball serving, in positions at the extreme range of motion, increasing the risk of shoulder or other upper limb injury ( Paraskevopoulos *et al.*, 2021a, 2021b, 2023). Nevertheless, the increased physical demands placed at the shoulder and elbow in overhead athletes may also affect physical performance during play ( Clarsen *et al.*, 2015). Despite the presence of symptomatology in this population, research has shown that they continue to train and compete irrespective of clinical symptom severity ( Agresta *et al.*, 2019; Clarsen & Bahr, 2014). Experts in the field support that prevention is key in the management of overhead athletes, including evaluation and monitoring of upper limb health and performance ( Cools *et al.*, 2015; Sukanen *et al.*, 2022). Patient-reported outcome measures (PROM) are highly suggested in this clinical population for the assessment of functional performance ( Paraskevopoulos *et al.*, 2023).

The Kerlan-Jobe Orthopedic Clinic (KJOC) Shoulder and Elbow Score has shown to be a valid and reliable PROM that can be used specifically in overhead athletes ( Alberta *et al.*, 2010; Merolla *et al.*, 2018). The KJOC was designed by Alberta *et al.* (2010) for the assessment of the functional status of the upper limb of patients involved in highly demanding overhead sports. The KJOC includes questions related to self-perceived ability to perform sport-specific movements such as, hitting a ball. The KJOC has been designed not only for symptomatic athletes but also for the assessment of the functional performance of healthy athletes as well ( Sukanen *et al.*, 2022). Lastly, the KJOC can also be used in order to examine the effectiveness of any intervention in overhead athletes with shoulder or elbow pathology ( Sukanen *et al.*, 2022).

The original version of the KJOC has been translated from English into several other languages, including Korean ( Oh *et al.*, 2017), Finish ( Sukanen *et al.*, 2022), Italian ( Merolla *et al.*, 2017), Turkish ( Turgut & Tunay, 2018), Norwegian ( Fredriksen & Myklebust, 2019) and German ( Schulz *et al.*, 2022). To date no comparable PROM exists in the Greek language, although overhead sports are highly popular in Greece. Thus, the aim of this study is to translate and cross culturally adapt the KJOC in Greek-speaking overhead athletes and examine the reliability and validity of the Greek version of the KJOC in this population.

## Protocol

The authors will collect data from multiple outpatient private clinics in Greece. To be eligible to participate in the study patients should meet the following criteria: 1) be adults (aged >18 years), 2) to compete and train at least two times weekly in an overhead sport, such as handball, volleyball, baseball, softball, basketball, water polo, tennis, or badminton and 3) to be able to natively communicate, read and write in Greek. Healthy and injured players with shoulder or elbow complaints will be included. Athletes will be excluded if they suffer from cognitive, communication, or psychological issues. Also, athletes suffering from neurological dysfunction or cardiovascular or pulmonary dysfunctions that result in functional limitations will be excluded. All participants will sign an informed consent form before participation. The study protocol has been approved by the ethics committee of the University of Peloponnese.

### Sample size

A minimum sample of 100 participants will be considered adequate for the assessment of the internal consistency, floor and ceiling effects, construct validity, test-retest reliability, and measurement error based on the recommendation of the consensus-based standards for the selection of health measurement instruments (COSMIN) ( Terwee *et al.*, 2012).

### Translation and cross-cultural adaptation

The procedures for translation and cross-cultural adaptation of the KJOC will be based on previously reported studies ( Beaton *et al.*, 2000; Schulz *et al.*, 2022). The first step includes the forward translation of the English version into Greek by two independent bilingual translators who are Greek in origin. One of the two forward translators is a Lecturer in physiotherapy with more than 30 years of experience in clinical physiotherapy and academic teaching. The other forward translator will have no physiotherapy or medical background and will be unaware of the existence of the KJOC. The two forward translators will compare their translations until a consensus is reached. A single Greek version of the scale will be formed from the two reports and the comments of the two translators. Again, another two bilingual independent translators will complete the two backward translations of the Greek version of the KJOC into English. The backward translators will compare the scale with the translated version in order to confirm whether the semantic, conceptual, and experimental equivalence are met.

The pre-final Greek version of the KJOC will be then pilot tested in 10 overhead athletes. From these 10 athletes, five will be symptomatic and five will be asymptomatic in the shoulder or elbow region. After completion of the 10 pilot pre-final versions of the KJOC an interview will be followed individually with all 10 athletes. Two physiotherapists with more than 10 years of experience will conduct the interviews. In each individual interview the participants will be asked to explain if the content of the pre final version of the scale is clear after reading the instructions, the items and responses. Furthermore, they will be asked to clarify whether parts of the scale are not clear and will provide suggestions for any possible modifications that may improve clarity.

### Reliability

Test-retest reliability will be examined in the final Greek Version of the KJOC (Gr-KJOC). All participants (n=100) will be asked to complete the Gr-KJOC twice. The first time that they will complete the Gr-KJOC will be during their first contact with the authors and the second will be three to five days later. The interval of test-retest sessions in this study is specified in order to minimize recall and to be suitably short to guarantee clinical stability between testing sessions ( Aljathlani *et al.*, 2022). The clinical stability of the participants will be examined by asking each one whether they believed that their symptoms were the same in the retest session ( Bennell *et al.*, 2000). Only patients that answer that their symptoms are the same in the retest session will complete the Gr-KJOC for a second time. Completion of the Gr-KJOC twice should allow the investigators to examine test-retest reliability by comparing the results of the test and retest sessions. Internal consistency will be also assessed based on the degree to which separate items of the Gr-KJOC relate to each other ( English & Keeley, 2014).

### Validity

The construct validity will be examined after correlating the results of the Gr-KJOC with the Greek Version of the Disability of the Arm, Shoulder, and Hand (DASH) and the DASH Sports Module (DASH-SM) ( Aljathlani *et al.*, 2022). The Greek DASH has shown to be a reliable and valid instrument that can provide a standardized measure of patient-centred outcomes in Greek-speaking patients with upper limb disorders while the DASH-SM assesses symptoms and the functional status of the upper limb in sports settings ( Themistocleous *et al.*, 2006). The DASH contains 30 questions; 21 related to function, six related to symptom severity and three to social function. Each question is rated on a 5-point scale (1, no difficulty; 2, mild difficulty; 3, moderate difficulty; 4, severe difficulty; 5, unable). The questionnaire score is calculated by applying established formulas for the first 30 questions and the scores range from 0 (the best) to 100 (the worst) ( Themistocleous *et al.*, 2006). The DASH-SM contains four questions and the goal of the DASH-SM is to identify the specific difficulties that athletes might experience but which may not affect their activities of daily living and consequently may go “undetected” in the 30-item portion of the DASH ( Aljathlani *et al.*, 2022).

### Feasibility

The mean duration for reading and completing the questionnaire by the athletes will be measured. Also, the duration needed for analysing the results of the questionnaire by one of the investigators and calculating the final score will be also measured. Questionnaire analysis will be conducted by one investigator in all cases.

### Floor and ceiling effect

Verification of floor and ceiling effect will be made by percentage (>15%) of the participants who have obtained the minimum and maximum scores in the Gr-KJOC, respectively ( Terwee *et al.*, 2007).

### Statistical analysis

All data will be analysed using IBM SPSS statistics (RRID:SCR_016479) 28.0. Descriptive statistics will be calculated and reported for all measures. The statistical level of significance will be set at p < 0.05. Normal distribution of the data will be examined *via* the Shapiro-Wilk test. Parametric tests will be selected for data with normal distribution and non-parametric tests will be selected for data without normal distribution.

*Internal consistency*

Internal consistency, as a degree of homogeneity of the single items of the Gr-KJOC, will be examined using Cronbach’s alpha. Internal consistency will be considered acceptable for the Gr-KJOC if the alpha value is going to be within the recommended range of 0.70 to 0.95 ( Aljathlani *et al.*, 2022; Prinsen *et al.*, 2018).

*Test re-test reliability (measurement errors)*

The Intraclass Correlation Coefficient (ICC) for absolute agreement will be used to examine test re-test reliability of each item and the total score of the Gr-KJOC. Measurement errors from the use of Gr-KJOC will be estimated from the Standard Error of Measurement (SEM) and the Minimum Detectable Change (MDC). For the SEM the following equation will be used: SEM = SD√(1-ICC). SD will be the pooled SD calculated by the following equation: SDpooled = √(SD1 ^2^ + SD2 ^2^)/2. Then, the MDC will be calculated using the following equation: MDC= SEM × 1.64 × √ 2 reflecting the smallest detectable within-person change in score ( Aljathlani *et al.*, 2022; Portney & Watkins, 2009; Valentín-Mazarracin *et al.*, 2021).

*Construct validity*

Construct validity of the Gr-KJOC will be examined by correlating the results of the Gr-KJOC with the DASH and the DASH-SM. Spearman’s correlation will be used to examine construct validity. Correlation coefficients of 0.70-0.89, 0.40-0.69 and 0.10-0.39 will be considered as strong, moderate and weak, respectively ( Schober *et al.*, 2018).

The known group method analysis will also be used to assess a further aspect of construct validity and identify whether the Gr-KJOC can actually differentiate between symptomatic (playing with arm symptoms or unable to play due to arm symptoms) and asymptomatic athletes (playing without any arm symptoms), as previously performed ( Schulz *et al.*, 2022). In order to establish evidence of known-group validity, independent samples t-test and Mann–Whitney U test will be used. Also, Pearson’s effect size r will be calculated.

## Conclusions

This study will translate and culturally adapt the original KJOC scale into Greek for the first time using a standardized approach. We aim to show that the Greek version of the KJOC will be reliable and valid to use for the assessment of the functional capacity of healthy and injured overhead athletes in the should or elbow. Our aim through this research process will be to develop a translated KJOC that will be used by researchers and health care professionals, as well as coaches in Greek-speaking athletic settings for assessing functional status, treatment outcome and return-to-sport ability in this population. Lastly, the KJOC can also be used to predict shoulder or elbow injuries since there is evidence to show that there is a relationship between lower KJOC scores and future in-season injuries for overhead athletes ( Holtz & O’Connor, 2018).

### Ethics

The study protocol was approved by the Institutional Ethics Committee of the University of Peloponnese (Ref No. 7365).

### Study status

The study has started. The study has not been completed and no data analysis has been performed.

## Data Availability

No data are associated with this article.
