# Supplementary material for: Cross-cultural adaptation and validation of the Greek version of the Kerlan-Jobe orthopaedic clinic shoulder and elbow score in Greek overhead athletes
Source: F1000Res. 2023 May 15;12:498. [Version 1] doi: 10.12688/f1000research.134195.1 (PMC11301131; doi:10.12688/f1000research.134195.1)
Supplement: Peer review history

# Reviewer report: Reviewer response for version 1

**DOI:** 10.5256/f1000research.147227.r305665

**Published:** 2024-08-05

**Authors:** Maria Sukanen

**Affiliations:** 1 University of Jyväskylä, Jyväskylä, Finland

The authors report a study protocol about cross-cultural adaptation and validation process of the Kerlan-Jobe orthopaedic clinic shoulder and elbow score in Greek language. The protocol shows the efforts the authors have made with planning the research according to accepted standards. The protocol is mostly very clearly presented and below I have listed some minor comments and recommendations for the author’s consideration.

- In the abstract (methods), I would replace the term *ground* with *floor*, as later on in the manuscript you refer to floor effect.
- In the abstract (conclusions), I recommend adding that this protocol also describes the assessment of psychometric properties of the Greek KJOC in addition to cross-cultural adaptation process.
- Many of the keywords are included in the study title, perhaps I would consider replacing e.g., shoulder, elbow and KJOC for example with psychometric properties and reliability.
- In the introduction you can add that KJOC has also been validated into Spanish language. Please also note that Finnish is written with two n’s.
- In the end of introduction, I would rephrase the study aim more precisely to match this study protocol.
- Under the subheading “Sample size”, it is perhaps not necessary to introduce the abbreviation COSMIN.
- Under the subheading “Translation and cross-cultural adaptation”, did only the translators work through the adaptation process of the Greek KJOC? Many times, multidisciplinary team input is of great value during cross-cultural adaptation.
- Under “Reliability” I would suggest extending the test-retest time interval closer to a week.
- In general, regarding the so far reported measurement properties of KJOC, the authors could briefly conclude what kind of validity/reliability results previous studies have obtained for the same variables you report that will be tested for the Greek KJOC.
- Under "validity", the authors provide general information regarding DASH as a comparison score, but this information is lacking for KJOC. I would recommend the authors to add a description of KJOC as well.
- Under “validity”, it is a great advantage that the authors have the possibility to use a translated and validated questionnaire as a comparison score to test validity.
- I would recommend the authors to follow the same order while describing measurement properties throughout the manuscript (abstract, data collection, analysis) e.g., starting with internal consistency, followed by validity, reliability, floor- and ceiling effect etc. This would make the storyline more logical for the reader.
- I would also add information of obtaining permission to move forward with the study from the original developer of the KJOC score.

Is the study design appropriate for the research question?

Yes

Is the rationale for, and objectives of, the study clearly described?

Partly

Are sufficient details of the methods provided to allow replication by others?

Yes

Are the datasets clearly presented in a useable and accessible format?

Not applicable

Reviewer Expertise:

My area of research lies between sport and health sciences with a background in physiotherapy. I have expertise in conducting cross-cultural adaptation and validation research of patient-reported outcomes from a previous publication and current studies.

I confirm that I have read this submission and believe that I have an appropriate level of expertise to confirm that it is of an acceptable scientific standard.

# Reviewer report: Reviewer response for version 1

**DOI:** 10.5256/f1000research.147227.r263359

**Published:** 2024-05-22

**Authors:** Julia Fayão

**Affiliations:** 1 University of Sao Paulo Ribeirão Preto Medical School, Sao Paulo, Brazil

I recommend extending the reliability testing time to up to seven days instead of five

 I suggest conducting responsiveness testing.

 It is important to mention whether there was authorization from the original author for the translation and validation.

 I recommend providing more information about the questionnaire that will be translated and validated into Greek.

 I suggest including "study protocol" in the title.

 As for my thoughts on the article, I would like to highlight its relevance in the context of cultural adaptation and validation of questionnaires for Greek high-performance athletes. This type of study is crucial to ensure the accuracy and reliability of measurements across different cultures and athletic contexts. Organizing my comments into 'Major' and 'Minor' points, I would like to address methodology, clinical relevance, and suggestions for improvement:

 Major:

 The methodology used for translation and validation is essential to ensure conceptual and linguistic equivalence of the questionnaire.

 The clinical relevance of this study lies in the need for specific assessment tools for Greek athletes due to the unique demands of their sports.

 The study has the potential to positively impact clinical practice and research in sports medicine by providing a validated and culturally adapted assessment tool.

 Minor:

 It would be helpful to provide more details on the specific methods used in translating and validating the questionnaire.

 A discussion of the study limitations and possible areas for future research would be valuable to contextualize the results.

 Thank you again for the opportunity to review this study.

Is the study design appropriate for the research question?

Yes

Is the rationale for, and objectives of, the study clearly described?

Yes

Are sufficient details of the methods provided to allow replication by others?

Yes

Are the datasets clearly presented in a useable and accessible format?

Yes

Reviewer Expertise:

My doctoral research consists of an assessment of the shoulder complex in volleyball athletes

I confirm that I have read this submission and believe that I have an appropriate level of expertise to confirm that it is of an acceptable scientific standard.
